# Prevalence and determinants of depression among pharmacy students enrolled in a newly implemented pharmd curriculum in Egypt: a cross-sectional study

**DOI:** 10.1186/s40359-025-03370-z

**Published:** 2025-09-17

**Authors:** Mohamed Hendawy, Mohamed Abouzid, Aliaa Gamal, Marwa Elnagar, Mostafa Hossam El Din Moawad

**Affiliations:** 1 Faculty of Pharmacy, Alexandria University, Alexandria, Egypt; 2 Department of Physical Pharmacy and Pharmacokinetics, Faculty of Pharmacy, Poznan University of Medical Sciences, Rokietnicka 3 St, Poznan, 60-806 Poland; 3 Doctoral School, Poznan University of Medical Sciences, Poznan, 60-812 Poland; 4 Clinical Pharmacy Department, Faculty of Pharmacy, Deraya University, Minia, Egypt; 5 Faculty of Pharmacy, MTI University, Cairo, Egypt; 6 Alexandria Main University Hospital, Alexandria, Egypt; 7 Faculty of Medicine, Suez Canal University, Ismailia, Egypt

**Keywords:** Depression, Pharmacy students, Egypt, PharmD program, Mental health, Socioeconomic status, Academic stress

## Abstract

**Background:**

Depression is a prevalent mental health concern among university students, particularly those in healthcare fields like pharmacy. The introduction of the Doctor of Pharmacy (PharmD) program in Egypt has brought new academic and professional pressures, making it critical to evaluate its impact on students’ mental well-being.

**Methods:**

A cross-sectional study was conducted among 576 PharmD students in Egypt using an online survey. The survey collected demographic information and assessed depression levels using the Beck Depression Inventory (BDI). Statistical analyses, including Mann-Whitney U tests and logistic regression, were used to identify predictors of depression.

**Results:**

The study found that 40.5% of participants exhibited borderline to moderate depression, while 24.7% displayed significant depressive symptoms. Key predictors of higher depression scores included socioeconomic status and type of university. Students attending public universities had significantly higher depression scores compared to those in private institutions (*p* = 0.033). Financial strain was strongly associated with higher depression levels, with students experiencing insufficient funds throughout the year showing the highest depression scores. Other demographic factors, such as sex, residence, and family involvement in healthcare, were not significantly associated with depression levels.

**Conclusion:**

Depression is highly prevalent among PharmD students in Egypt, particularly those facing financial challenges or attending public universities. These findings underscore the urgent need for targeted mental health interventions and financial support programs to alleviate the psychological burden on pharmacy students.

## Introduction

Depression is one of the most prevalent mental health disorders all over the world, influencing approximately 280 million people [1]. It is distinguished by persistent sadness, loss of interest, and impaired daily functioning, potentially leading to more severe consequences, such as suicide. Among university students, mental health challenges are pronounced, with depression being a noteworthy issue. The demanding academic environment, particularly in health-related disciplines such as pharmacy, increases the risk of depression among students [2].

Pharmacy students face a unique set of academic and emotional pressures. The curriculum is strict, with high expectations for academic achievement, extensive clinical training, and future professional responsibilities. As these students strive to balance their academic workload with their personal and social lives, mental health issues such as depression often emerge [3]. Research from around the world consistently shows that students in health-related fields, such as pharmacy, tend to experience higher levels of depression and anxiety compared with their peers in other disciplines [4]. Despite this global pattern, there is a lack of focused studies on depression among pharmacy students in Egypt, particularly following the introduction of the new Doctor of Pharmacy (PharmD) program.

Implementing the PharmD degree in Egypt marks a substantial shift in pharmacy education. Traditionally, pharmacy students in Egypt completed a Bachelor of Pharmacy (BPharm), emphasizing pharmaceutical sciences, with a decreased focus on clinical practice. However, the PharmD program mirrors international standards by incorporating more patient-centered training, clinical decision-making, and direct patient care into the curriculum [5]. While this transition aims to better prepare students for contemporary healthcare demands, it has introduced additional academic and professional pressures. Increased clinical rotations, patient interactions, and the need to meet new competencies can heighten stress and contribute to mental health issues, particularly depression [6]. The mental well-being of students enrolled in the PharmD program is thus a critical area for research.

A growing body of evidence highlights the prevalence of depression among healthcare students [7]. This trend aligns with global findings, showing that pharmacy students often experience higher levels of depression compared with those in other academic disciplines due to the combination of academic stress, uncertainty regarding future career prospects, and the intensive nature of clinical training [8].

However, despite increased awareness of mental health issues in healthcare professions, limited research has evaluated how the newly implemented PharmD program in Egypt affects the mental well-being of pharmacy students. Given the shift towards a clinically oriented education model, it is essential to examine whether this new program exacerbates or alleviates the mental health challenges these students face. Understanding the prevalence of depression within this context is vital for informing educational policies and mental health support systems tailored to the needs of PharmD students.

This study aims to evaluate the prevalence of depression among pharmacy students in Egypt under the new PharmD program. Through a cross-sectional survey, the research will provide critical insights into the mental health status of pharmacy students, identify key stressors, and explore the risk factors contributing to depression. The outcomes will be valuable for developing targeted interventions to improve student mental health, enhance academic performance, and promote professional resilience. This is essential for cultivating a generation of pharmacists skilled in clinical practice and equipped to maintain their mental well-being.

## Methods

### Study design

Following the Checklist for Reporting Results of Internet E-Surveys (CHERRIES) [9], we performed a cross-sectional study. We used an anonymous, self-administered online survey tool through the “Microsoft Forms” platform.

### Study population

We conducted a survey involving pharmacy students across various faculties in Egypt, specifically focusing on those enrolled in the newly implemented PharmD program. The inclusion criteria for participants were as follows: being at least 18 years old, currently enrolled as a full-time PharmD student in any Egyptian pharmacy school, and having completed at least one academic year in the program. No restrictions were imposed based on sex, year of study, university, or socioeconomic status. Students under 18 years of age, enrolled in non-PharmD programs, or who did not provide consent to participate were excluded from the study. Furthermore, those who submitted incomplete or inaccurate survey responses were excluded from the final analysis.

### Sampling

Once we created the survey, we initially distributed it to pharmacy students who met the inclusion criteria from August 2024 to October 2024. We then asked them to share it with their colleagues -specifically PharmD students- to create a snowball effect and maintain the inclusion criteria. Additionally, to reach the target audience further, we shared the survey through professional and social media channels such as LinkedIn, Facebook, and WhatsApp. In some cases, invitations were also extended after classes with the approval of the faculty. We clearly stated that the survey was intended only for PharmD pharmacy students who met the specific inclusion criteria. To verify eligibility, we aimed to include all 27 Egyptian governorates (Alexandria, Aswan, Asyut, Beheira, Beni Suef, Cairo, Dakahlia, Damietta, Faiyum, Gharbia, Giza, Ismailia, Kafr El Sheikh, Luxor, Matrouh, Minya, Monufia, New Valley, North Sinai, Port Said, Qalyubia, Qena, Red Sea, Sharqia, Sohag, South Sinai, Suez).

OpenEpi was used to calculate the sample size. Assuming the prevalence of anxiety and depression of 51% (*P* = 0.51), similar to that reported by Elnaem et al. 2022 [10], with a 95% confidence level (Z = 1.96) and 5% margin of error (E = 0.05), approximately 384 students were required to detect a similar prevalence rate, calculated as follows:

$$A\, = \,{Z^2}\, \times \,P\, \times \,\left( {1\, - \,P} \right)\,/\,{E^2}$$

### Study tool

We divided the survey into two sections: (1) demographic characteristics (age, sex, governorate, academic year, living in a rural or urban place, being the first generation pharmacist in the family, having a plan before coming into pharmacy school, having any of your family members working in health care sector, studying in a private or public university, socioeconomic status and Primary Source of Information related to pharmacy related career); (2) Beck Depression Inventory (BDI), 21-item, self-report rating inventory that measures characteristic attitudes and symptoms of depression [11]. Each item was scored 0 to 3 points for a total score range of 0 to 63. According to the OHSU Headache Center [12], Beck’s criteria for depression levels classify scores of 1 to 10 as normal, 11 to 16 as mild, 17 to 20 as borderline clinical depression, 21 to 30 as moderate depression, 31 to 40 as severe depression, and over 40 as extreme depression.

### Ethical consideration

We conducted this study according to the Declaration of Helsinki. Written informed consent was obtained electronically from all participants. Participation in the online survey was entirely voluntary; participants could exit the survey at any time without penalty. Additionally, all responses were reported and analyzed anonymously to ensure participants’ confidentiality throughout the research process. Ethical approval was obtained from the Faculty of Pharmacy, Deraya University, Minia, Egypt.

### Statistical analysis

We performed the statistical analysis using PQStat v.1.8.2.238. Pairwise deletion was applied to manage missing data. The validity of the BDI was calculated using standardized Cronbach’s Alpha. The Shapiro–Wilk test assessed the normality of continuous variables. Categorical variables are presented as frequency and percentage, while continuous variables are reported as mean and standard deviation (SD) for normally distributed data or median and interquartile range (IQR) for non-normally distributed data. The Mann–Whitney U test was used to compare total depression scores across sex, living location, type of university, first-generation pharmacy status, career planning, family involvement in healthcare, and financial status.

Differences in depression severity levels (mild, moderate, and significant) by financial status categories (e.g., insufficient funds all year, occasional insufficiency, balanced funds, sufficient most of the time) were evaluated with Kruskal-Wallis ANOVA, followed by Dunn-Bonferroni post hoc tests. Spearman’s rank correlation coefficient (r) examined the relationships between total depression scores and continuous variables, including age and academic year. A multivariable logistic regression model was developed to analyze associations with financial status and university type, alongside univariate analyses to examine predictors such as sex, age, academic year, living place, first-generation status, career plans, family in healthcare, socioeconomic status, and university type. Additionally, univariate and backward stepwise logistic regression models identified predictors of severe to extreme depression (i.e., significant depression, defined as a total depression score of ≥ 31). Results are presented as odds ratios (ORs) with 95% confidence intervals (CIs), and a p-value < 0.05 was considered statistically significant across all tests.

## Results

### General characteristics

A total of 576 PharmD students completed the survey. The standardized Cronbach’s alpha was 0.929, indicating excellent internal consistency [13]. The average age of participants was approximately 21.5 years, with a majority being female (67.9%). Most participants resided in urban areas (60.4%), and a considerable portion were first-generation pharmacists (72.9%).

Regarding their plans before attending pharmacy school, 56.8% did not have a plan. About half of the participants had family members working in the healthcare sector (47.6%). Socioeconomic status varied, with 60.4% having balanced funds, while a small percentage (3.3%) reported insufficient funds for the year.

**Fig. 1:**
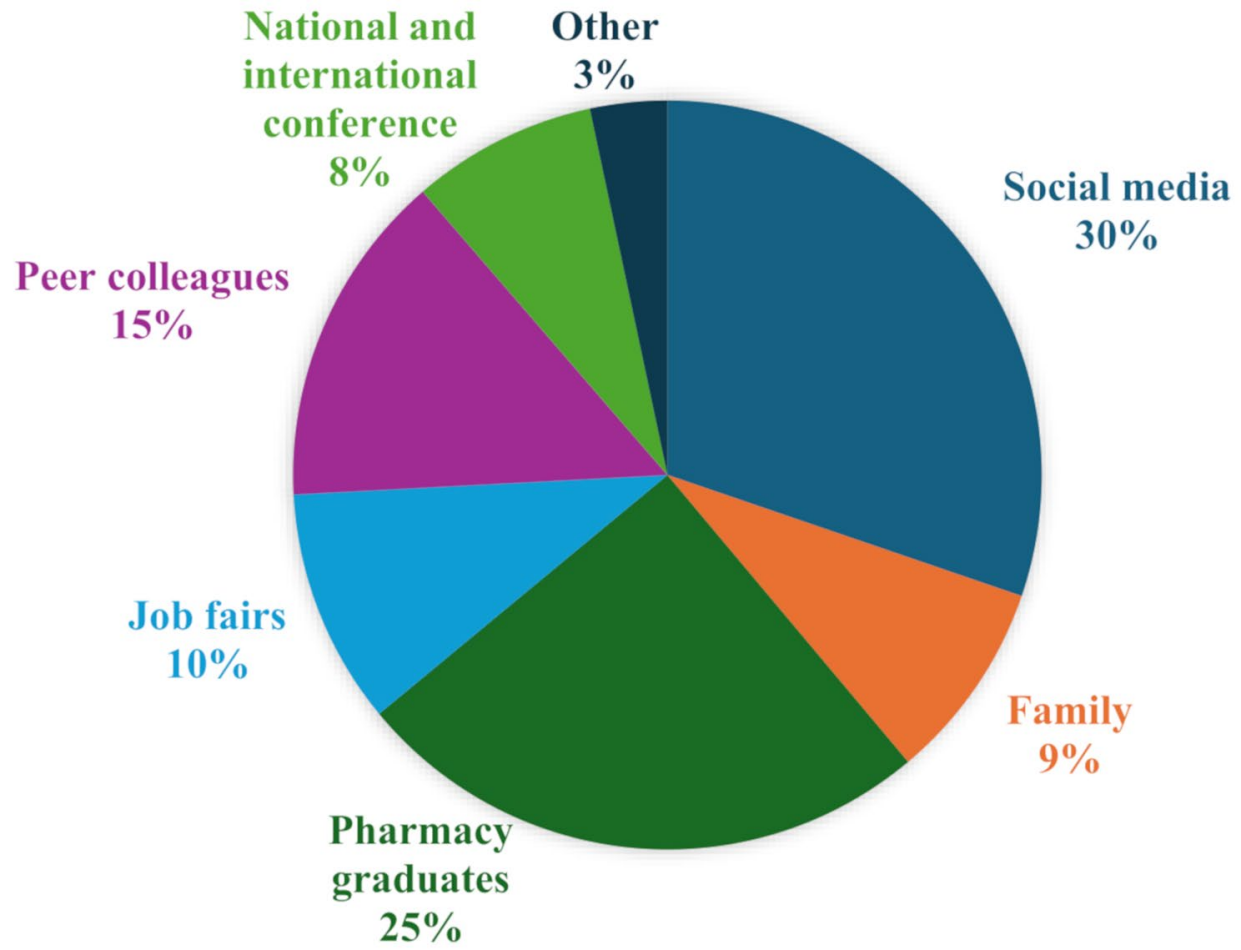
Source of information related to pharmacy career

Sources of information about pharmacy careers were diverse, with social media being the most common (336 responses), followed by pharmacy graduates (279 responses) (Fig. 1). Most participants (54%) attended public universities and were in their last three years of academic program (68.2%).

Regarding mental health, 40.5% of participants had borderline to moderate depression scores, while 24.7% had severe to extreme depression scores (Table 1).

**Table 1:** Demographic characteristics of pharmd students who participated in the survey

Categorical Variable, *n* (%)	
**Age**	21.53 ± 2.16; 22 (20–23)*
**Sex**	
Female	391 (67.9)
Male	185 (32.1)
**Residency**	
Rural	228 (39.6)
Urban	348 (60.4)
**First-generation pharmacist**	
No	156 (27.1)
Yes	420 (72.9)
**Having a plan before coming to pharmacy school**	
No	327 (56.8)
Yes	249 (43.2)
**Family members working in the healthcare sector**	
No	302 (52.4)
Yes	474 (47.6)
**Socioeconomic status**	
Insufficient funds for whole years	19 (3.3)
Insufficient funds for some times	95 (16.5)
Balanced funds	348 (60.4)
Sufficient funds for most times	114 (19.8)
**Type of university**	
Private	265 (46)
Public	311 (54)
**Academic year**	
First three years	184 (31.8)
Last three years	392 (68.2)
Total depression score (BDI)**	
Normal/Mild (1–16)	185 (32.1)
Borderline/Moderate (17–30)	233 (40.5)
Severe/Extreme (≥ 31)	142 (24.7)

* mean ± standard deviation; median (interquartile range)

** 16 students (2.7%) had a total score of 0

### Differences between total depression scores according to demographic characteristics

Students attending public universities had significantly higher depression scores compared with those in private universities, with median scores of 22 (IQR: 13.5–32) versus 20 (IQR: 12–29), respectively (*p* = 0.033). Additionally, there was a significant difference in depression scores based on financial status (*p* < 0.001). Those with insufficient funds for the whole year had the highest median score of 35 (IQR: 21-49.5), while those with sufficient funds most times had the lowest median score of 19 (IQR: 11–29) (Table 2).

No significant differences were found in depression scores according to sex, residency, generation, having plans before attending pharmacy school, or having family members working in the healthcare sector (Table 2). Furthermore, age and academic year showed insignificant weak correlations with the total depression score (*r* < 0.001, 95% CI: -0.083 to 0.085, *p* = 0.981) and (*r* = -0.22, 95% CI: -0.106 to 0.062, *p* = 0.592), respectively.

**Table 2:** Differences between total depression scores according to demographic characteristics

Comparator	*n*	Depression, median (IQR)	*p*-value
Sex			
Male	185	20 (12–31)	0.413
Female	391	21 (13–30)	
Living place			
Rural	228	22 (12.75-31)	0.565
Urban	348	20 (13–30)	
Type of university			
Public	311	22 (13.5–32)	0.033*
Private	265	20 (12–29)	
First generation pharmacist			
Yes	420	21 (13–30)	0.78
No	156	20 (12.75-31)	
Plans			
Yes	249	21 (13–30)	0.895
No	327	21 (12–30)	
Family working in healthcare			
Yes	274	20 (12–30)	0.456
No	302	21 (13–31)	
Finance status**			
Insufficient funds for whole years	19	35 (21-49.5)^a^	< 0.001*
Insufficient for some times	95	26 (17.5–35.5)^b^	
Balance	348	20 (12.75-29)^ab^	
Sufficient funds for most times	114	19 (11–29)^ab^	

* Statistically significant

** a and b denote the presence of significant differences between sub-groups holding the same letter, according to POST-HOC (Dunn Bonferroni),

### Multivariate model for total BDI scores with demographic characteristics

**Fig. 2:**
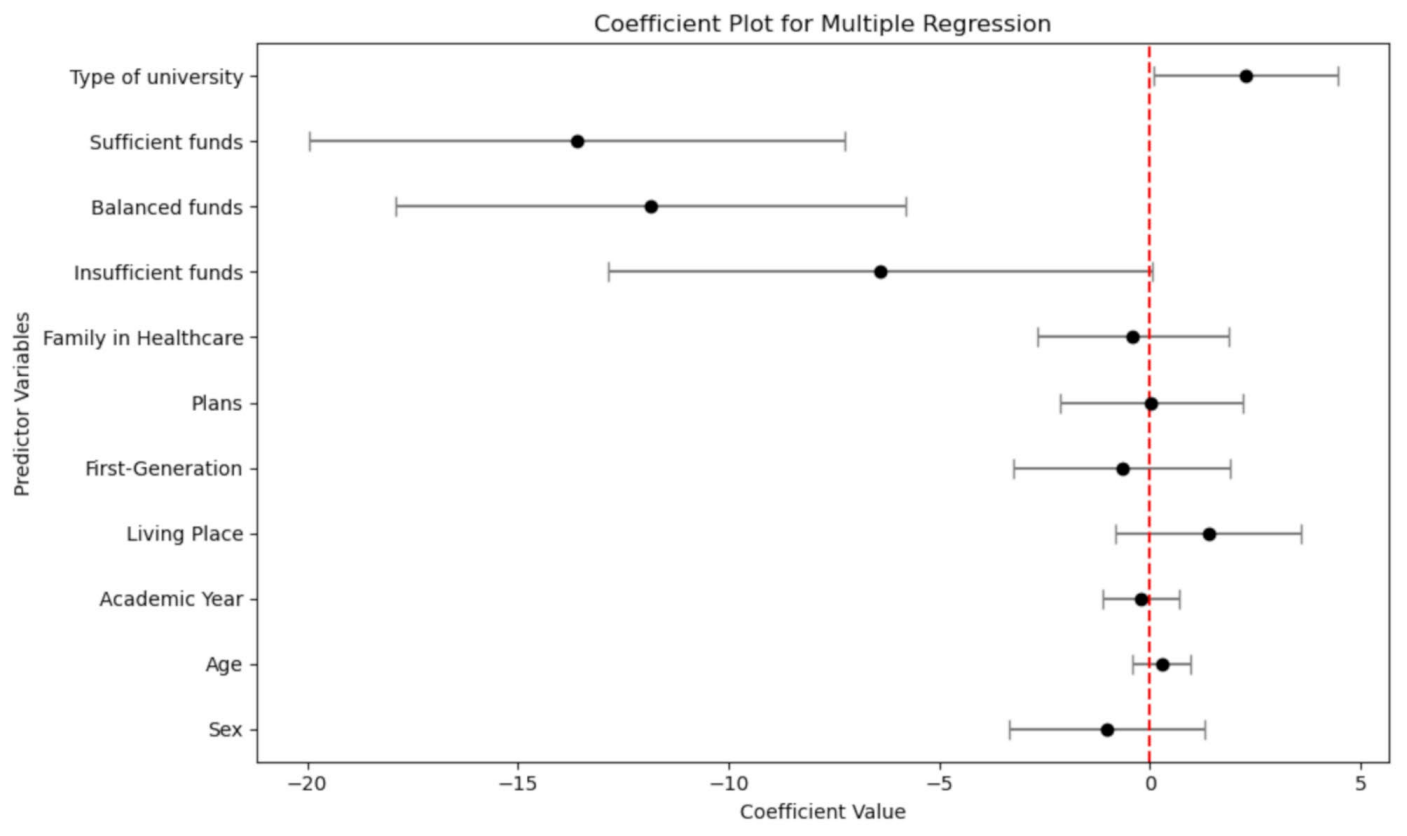
Multivariate model for total BDI scores with demographic characteristics

In the multivariate model, only socioeconomic status and type of school attendance showed significant results. Concerning socioeconomic status, there was a trend toward less total depression as long as funds were available. For example, compared with insufficient funds throughout the year, those who had sufficient funds had 13.6 fewer points (95% CI -19.96 to -7.24, *p* < 0.001), and 11.9 fewer points for those with balanced funds (95% CI -17.91 to -5.8, *p* < 0.001). Students attending public universities had 2.28 points higher in depression than those attending private universities (95% CI 0.102 to 4.449, *p* = 0.04) (Fig. 2).

### Predictors of significant depression (total BDI ≥ 31)

In the univariate predictor model for total BDI ≥ 31, students with balanced and sufficient funds were approximately 80% less prone to significant depression, with values of (OR 0.186, 95% CI 0.072 to 0.481, *p* < 0.001) and (OR 0.194, 95% CI 0.070 to 0.536, *p* = 0.002). Additionally, being in public universities increased the chances of developing significant depression by 60% (OR 1.60, 95% CI 1.087 to 2.365, *p* < 0.001). It is worth noting that these three factors were also retained in the multifactorial backward stepwise logistic regression model (Table 3).

**Table 3:** Predictors of significant depression (total BDI ≥ 31)

	b coeff.	b error	-95% CI	+ 95% CI	Wald stat.	*p*-value	odds ratio	-95% CI	+ 95% CI
Univariate predictors									
Sex (male)	0.059	0.206	-0.344	0.463	0.083	0.773	1.061	0.709	1.589
Age	-0.020	0.047	-0.112	0.072	0.178	0.673	0.980	0.894	1.075
Academic year	-0.039	0.061	-0.159	0.081	0.405	0.525	0.962	0.853	1.084
Living place (urban)	0.126	0.199	-0.264	0.517	0.402	0.526	1.135	0.768	1.677
1st generation (yes)	-0.072	0.216	-0.495	0.351	0.112	0.737	0.930	0.609	1.420
Plans (yes)	-0.015	0.195	-0.397	0.368	0.006	0.940	0.985	0.672	1.445
Family in healthcare (yes)	-0.133	0.194	-0.514	0.247	0.472	0.492	0.875	0.598	1.280
Socioeconomic status (reference, insufficient funds for whole years)									
Insufficient for some times	-0.812	0.511	-1.813	0.188	2.533	0.112	0.444	0.163	1.207
Balanced funds	-1.680	0.483	-2.627	-0.732	12.079	0.001*	0.186	0.072	0.481
Sufficient funds for most times	-1.640	0.518	-2.656	-0.624	10.013	0.002*	0.194	0.070	0.536
Type of university (public)	0.472	0.198	0.084	0.861	5.671	0.017*	1.604	1.087	2.365
Multifactorial backward stepwise logistic regression model**									
Insufficient funds for some times	-0.832	0.514	-1.838	0.175	2.620	0.105	0.435	0.159	1.192
Balanced funds	-1.683	0.486	-2.636	-0.730	11.984	0.001*	0.186	0.072	0.482
Sufficient funds for most times	-1.607	0.521	-2.629	-0.585	9.505	0.002*	0.200	0.072	0.557
Type of university	0.447	0.203	0.049	0.845	4.856	0.028*	1.564	1.051	2.327

* Statistically significant

** When removing the insufficient for some times from the model, only school type remained (OR 1.6, 95% CI 1.09 to 2.37, *p* = 0.017), and this final model was significantly different from the presented one (Chi-Square 21.13, *p* < 0.001),

## Discussion

Our study revealed a considerable prevalence of depression among Egyptian pharmacy students and identified key determinants contributing to their mental health challenges. Among the participants, 40.5% exhibited borderline to moderate depression, while 24.7% displayed significant depressive symptoms. These findings are consistent with previous research reporting high levels of psychological distress among healthcare students, underscoring the need for enhanced mental health support within academic settings [14–16]. For instance, an Iraqi study found that 45.9% of healthcare students exhibited depressive symptoms, with an additional 24.8% showing borderline levels [17]. In Lebanon, following the return to school post-COVID-19, 30.7% of pharmacy students experienced severe to extremely severe depression [18]. Similarly, a study among male medical students at the University of Bisha found a depression prevalence of 26.8% [19], while a systematic review across 13 Middle East and North African countries reported a pooled prevalence of 33.03% among healthcare workers between 2005 and 2020 [20]. Notably, the high prevalence of depression among PharmD students in our study is concerning, particularly given that earning a professional doctoral degree should offer advantages over a bachelor’s degree. We may suggest that if this advanced degree does not lead to financial gains or career progress, it may be perceived as a burden rather than an advancement. For instance, while individuals holding a PharmD degree in Europe and the United States may apply directly for a Doctor of Philosophy (PhD) program in some universities in Egypt, they are still required to complete a master’s degree first. Additionally, the number of pharmacies in Egypt has grown to nearly three times the global average. Recent estimates show Egypt has more than 75,165 pharmacies, with one pharmacy serving every 1,261 citizens, compared to the international average of one pharmacy per 3,500–5,000 individuals [21]. Due to market saturation, a 2018 cross-sectional survey of 1,500 pharmacists revealed that 55% of participants were considering a career shift [21].

In this study, socioeconomic status emerged as a prominent determinant, with students experiencing financial strain being more likely to report higher depression scores. Specifically, students with “insufficient funds for the whole year” had the highest median depression score, while those with sufficient or balanced funds had notably lower scores. Economic insecurity often creates additional burdens beyond academic pressures. Studies conducted in the United States, including those by Adams et al. and Ryu and Fan, reported a consistent association between financial stress and mental health problems among college students, illustrating that financial stability serves as a protective factor against depression [22, 23]. Furthermore, financial challenges are particularly impactful among healthcare students, who may face higher educational expenses and more extended academic programs than in other fields. The demanding nature of pharmacy education and financial insecurity might contribute to a heightened sense of vulnerability and stress among students [22, 23]. Studies indicate that enhancements in financial aid are positively associated with the retention of first-generation students, whereas escalations in loan debt are directly linked to a higher probability of attrition [24–26]. A study revealed that the integration of scholarships with peer advising, structured study groups, and mentorship markedly enhances retention, perseverance, and degree completion rates [27]. A separate study revealed that individualized student coaching enhanced retention by 14% after two years of intervention and elevated graduation rates from 31 to 34% among the participating students [28]. Hence, universities should implement financial aid policies and supportive initiatives to enable low-income and first-generation students to fully engage in opportunities essential for academic success, such as on-campus living and extracurricular participation.

The present findings indicated that the type of university was a significant factor for depression, as students attending public universities reported higher depression scores compared to those at private institutions. Contrary to our results, however, Al-Khlaiwi et al. [29] found that anxiety, depression, and stress were higher among private university medical students in Saudi Arabia than among those enrolled in public universities. Financial burden, instructional quality, student population, future employment prospects, and institutional reputation influence students’ mental health. Recent studies have suggested a greater prevalence of depression, anxiety, and stress among private school students compared with their counterparts at public schools [30, 31]. The COVID-19 pandemic further exacerbated financial pressures on private medical students, while parental behavior and heightened academic demands—such as increasing tuition—have also been identified as potential contributors to deteriorating mental health. Furthermore, a misalignment between students’ academic aspirations and their actual circumstances may amplify stress. Consequently, close examination of student satisfaction and expectations is vital to mitigate stress and anxiety [29].

It is important to note that the factors identified in previous studies may not fully apply to the Egyptian context. For instance, the student loan system is not widespread in Egypt, suggesting that private university students or their families often pay tuition without relying on loans. Additionally, the number of private universities in Egypt has grown steadily, particularly in the field of pharmacy. Reports indicate that enrollment in pharmacy faculties at private institutions has increased consistently over the past decade, with some documenting double-digit annual growth [32]. As the number of private universities rises, admissions chances become higher. These universities may also provide a more supportive environment than public institutions; for example, research has found that e-service quality, interactivity, comfort, and familiarity in private universities were positively related to student satisfaction and behavioral intentions [33, 34]. Although these factors may contribute to lower depression rates among PharmD students in private Egyptian universities, improvements are still needed. Previous work shows that public and private universities often fail to adopt student-centered teaching methods, incorporate quality assessment, and offer graduate job placement services [35].

In this study, demographic factors such as sex, residence (urban vs. rural), family involvement in healthcare, and prior career planning did not show significant associations with depression scores. For instance, no notable difference was observed between male and female students, indicating that these variables were not substantial predictors of depression levels in our analysis. This lack of sex-based disparity contrasts with findings from previous studies, where female healthcare students often reported higher levels of depression and anxiety due to perceived dual burdens of academic and familial responsibilities [36, 37]. Another study conducted in South Asia showed that undergraduate females are at increased risk of depression compared with males [38]. Pacheco et al. [39], in their meta-analysis, showed that females were at increased risk of depression.

Variables such as urban or rural residence and family involvement in the healthcare sector showed no significant correlation with depression scores, indicating that depression in this population is mainly independent of location or familial professional background. This lack of difference based on residence and family healthcare involvement may reflect the relatively homogeneous academic and social stressors experienced by pharmacy and medical students across diverse backgrounds [40, 41]. The overarching demands of pharmacy education, including rigorous coursework, clinical placements, and high-stakes exams, likely contribute to a uniform stress level for all students [42]. For instance, both urban and rural students face similar academic expectations and limited time for social activities, which may minimize any potential influence of residence on mental health. Similarly, while having family members in healthcare might offer initial familiarity with the field, it does not necessarily alleviate the academic pressures of pharmacy school. These universal stressors likely overshadow any advantage that background familiarity or location could provide.

While previous studies suggest that advanced students in healthcare fields often develop resilience over time, which is associated with decreased depression and better quality of life [43], our study found no significant association between depression levels and academic year. This finding may indicate that pharmacy students face consistent academic pressure across all years of study, perhaps due to the cumulative demands of their training program. Our findings align with the view that academic challenges among pharmacy students remain high throughout their program [38, 44], suggesting a need for consistent mental health support rather than interventions targeted solely at certain academic years.

### Strengths and limitations

This multi-institutional, CHERRIES-guided, cross-sectional online survey identified factors associated with depressive symptoms among PharmD students in Egypt using the 21-item BDI. Strengths include an adequate sample (*n* = 576), participation from both public and private universities across many governorates, and excellent internal consistency of the BDI (Cronbach’s α = 0.929). Important limitations should be noted. First, the cross-sectional design precludes causal inference and prevents establishing temporality. Second, all data were self-reported, which may introduce response and reporting biases; the BDI is a screening instrument rather than a clinical diagnosis.

Third, recruitment relied on convenience and snowball sampling via online channels and in-class invitations, which may lead to selection and coverage biases; consequently, generalizability is limited, and an exact response rate cannot be firmly established. Fourth, pairwise deletion was used for missing data, which can bias estimates if data are not missing completely at random. Fifth, while financial status showed strong associations with depression scores, we did not collect granular information on specific financial stressors, family income, or sources of financial support. Finally, potentially influential psychosocial variables (e.g., social isolation, anxiety, burnout, employment during study) were not measured, leaving room for residual confounding. These constraints should temper the interpretation of the observed associations.

## Conclusion

In this cross-sectional survey, 65.2% of Egyptian PharmD students reported borderline to significant depressive symptoms on the BDI. Lower financial resources and attendance at public universities were associated with higher total BDI scores and greater odds of significant depression (BDI ≥ 31). These findings indicate important correlates – not causes – of depressive symptoms. Future research should use longitudinal or mixed-methods designs, incorporate more granular financial measures, and examine additional psychosocial and academic factors (e.g., workload, social support, and institutional resources) to clarify temporality and mechanisms. Meanwhile, universities may consider implementing and prospectively evaluating comprehensive mental-health services, financial support programs, and targeted interventions – particularly in public institutions where higher depression scores were observed.

## Data Availability

No datasets were generated or analysed during the current study.

## Abbreviations

BDI: Beck Depression Inventory
CHERRIES: Checklist for Reporting Results of Internet E–Surveys
SD: Standard deviation
IQR: Interquartile range
ORs: Odds ratios
CIs: Confidence intervals

## References

[1] Depressive disorder (depression). https://www.who.int/news-room/fact-sheets/detail/depression. Accessed 4 Oct 2024.

[2] Ibrahim AK, Kelly SJ, Adams CE, Glazebrook C. A systematic review of studies of depression prevalence in university students. J Psychiatr Res. 2013;47:391–400. 10.1016/j.jpsychires.2012.11.015. 23260171 10.1016/j.jpsychires.2012.11.015

[3] Hussain R, Guppy M, Robertson S, Temple E. Physical and mental health perspectives of first year undergraduate rural university students. BMC Public Health. 2013;13:848. 10.1186/1471-2458-13-848. 24034822 10.1186/1471-2458-13-848 PMC3847612

[4] Dahlin M, Joneborg N, Runeson B. Stress and depression among medical students: a cross-sectional study. Med Educ. 2005;39:594–604. 10.1111/j.1365-2929.2005.02176.x. 15910436 10.1111/j.1365-2929.2005.02176.x

[5] Admissions R. & Prerequisites | LLU School of Pharmacy. https://pharmacy.llu.edu/admissions/requirements. Accessed 4 Oct 2024.

[6] El-Ibiary SY, Yam L, Lee KC. Assessment of burnout and associated risk factors among pharmacy practice faculty in the united States. Am J Pharm Educ. 2017;81:75. 10.5688/ajpe81475. 28630516 10.5688/ajpe81475 PMC5468713

[7] Agyapong-Opoku G, Agyapong B, Obuobi-Donkor G, Eboreime E. Depression and anxiety among undergraduate health science students: A scoping review of the literature. Behav Sci. 2023;13:1002. 10.3390/bs13121002. 38131858 10.3390/bs13121002 PMC10740739

[8] Pharmacy. and Medical Students’ Mental Health Symptoms, Experiences, Attitudes and Help-Seeking Behaviors - PMC. https://www.ncbi.nlm.nih.gov/pmc/articles/PMC6983890/. Accessed 4 Oct 2024. 10.5688/ajpe7558 PMC6983890 32001889

[9] Eysenbach G. Improving the quality of web surveys: the checklist for reporting results of internet E-Surveys (CHERRIES). J Med Internet Res. 2004;6:e34. 10.2196/jmir.6.3.e34. 15471760 10.2196/jmir.6.3.e34 PMC1550605

[10] Frontiers. | Assessment of mental wellbeing of undergraduate pharmacy students from 14 countries: The role of gender, lifestyle, health-related, and academic-related factors. https://www.frontiersin.org/journals/public-health/articles/10.3389/fpubh.2022.1011376/full. Accessed 4 Oct 2024. 10.3389/fpubh.2022.1011376 PMC9665407 36388263

[11] Beck AT, Ward CH, Mendelson M, Mock J, Erbaugh J. An inventory for measuring depression. Arch Gen Psychiatry. 1961;4:561–71. 10.1001/archpsyc.1961.01710120031004. 13688369 10.1001/archpsyc.1961.01710120031004

[12] Headache Diagnosis and Treatment | Brain Institute | OHSU. https://www.ohsu.edu/brain-institute/headache-diagnosis-and-treatment. Accessed 4 Feb 2025.

[13] Taber KS. The use of cronbach’s alpha when developing and reporting research instruments in science education. Res Sci Educ. 2018;48:1273–96. 10.1007/s11165-016-9602-2.

[14] Tsegay L, Ayano G. Psychological distress and associated factors among medical students in addis ababa, ethiopia: A cross-sectional study (May 2018). J Affect Disord Rep. 2024;16:100783. 10.1016/j.jadr.2024.100783.

[15] Sahu PK, Nayak BS, Rodrigues V, Umakanthan S. Prevalence of psychological distress among undergraduate medical students: A Cross-Sectional study. Int J Appl Basic Med Res. 2020;10:270. 10.4103/ijabmr.IJABMR_100_19. 33376702 10.4103/ijabmr.IJABMR_100_19 PMC7758794

[16] Hisato T, Nandy S, Monga EM, Sytek P, Abouzid M, Ahmed AA. Psychological distress among healthcare students in Poland from COVID-19 to war on ukraine: a cross-sectional exploratory study. Front Public Health. 2023;11. 10.3389/fpubh.2023.1186442. 10.3389/fpubh.2023.1186442 PMC10315478 37404286

[17] Kathem SH, Al-Jumail AA, Noor-Aldeen M, Najah N, Khalid DA. Measuring depression and anxiety prevalence among Iraqi healthcare college students using hospital anxiety and depression scale. Pharm Pract. 2021;19:2303. 10.18549/PharmPract.2021.2.2303. 10.18549/PharmPract.2021.2.2303 PMC8118599 34035869

[18] Fadel S, Fahda S, Akel M, Rahal M, Malhab SB, Haddad C, et al. Mental health assessment of Lebanese pharmacy students after returning to school post-COVID-19: A cross-sectional study. Pharm Educ. 2023;23:180–92. 10.46542/pe.2023.231.180192.

[19] Alshahrani AM, Al-Shahrani MS, Miskeen E, Alharthi MH, Alamri MMS, Alqahtani MA, et al. Prevalence of depressive symptoms and its correlates among male medical students at the university of bisha, Saudi Arabia. Healthcare. 2024;12:640. 10.3390/healthcare12060640. 38540604 10.3390/healthcare12060640 PMC10970630

[20] Abraham A, Chaabna K, Doraiswamy S, Bhagat S, Sheikh J, Mamtani R, et al. Depression among healthcare workers in the Eastern mediterranean region: a systematic review and meta-analysis. Hum Resour Health. 2021;19:81. 10.1186/s12960-021-00628-6. 34246282 10.1186/s12960-021-00628-6 PMC8271293

[21] Salem M, Ezzat SM, Hamdan D, Zayed A. Reorganization and updating the pharmacy education in egypt: A review study on the transition from B pharm to pharm D degree. J Adv Med Pharm Res. 2022;3:53–9. 10.21608/jampr.2022.152370.1043.

[22] Ryu S, Fan L. The relationship between financial worries and psychological distress among U.S. Adults. J Fam Econ Issues. 2022;44:16. 10.1007/s10834-022-09820-9. 35125855 10.1007/s10834-022-09820-9 PMC8806009

[23] Adams DR, Meyers SA, Beidas RS. The relationship between financial strain, perceived stress, psychological symptoms, and academic and social integration in undergraduate students. J Am Coll Health J ACH. 2016;64:362–70. 10.1080/07448481.2016.1154559. 10.1080/07448481.2016.1154559 PMC5086162 26943354

[24] Engle J, Tinto V. Moving beyond access: college success for Low-Income, First-Generation students. Pell Institute for the Study of Opportunity in Higher Education; 2008.

[25] Martin Lohfink M, Paulsen MB. Comparing the determinants of persistence for First-Generation and Continuing-Generation students. J Coll Stud Dev. 2005;46:409–28. 10.1353/csd.2005.0040.

[26] Somers P, Woodhouse S, Cofer J. Pushing the boulder uphill: the persistence of First-Generation college students. Naspa J. 2004. 10.2202/0027-6014.1353.

[27] Angrist J, Lang D, Oreopoulos P. Incentives and services for college achievement: evidence from a randomized trial. Am Econ J Appl Econ. 2009;1:136–63. 10.1257/app.1.1.136.

[28] Bettinger E, Baker R. The effects of student coaching in college: an evaluation of a randomized experiment in student mentoring. 2011. 10.3386/w16881

[29] Comparison of. depression, anxiety, and stress between public and private university medical students - PMC. https://pmc.ncbi.nlm.nih.gov/articles/PMC10451576/. Accessed 13 Nov 2024. 10.4103/jfmpc.jfmpc_1719_22 PMC10451576 37636173

[30] Sharma A, Sharma R. Internet addiction and psychological well-being among college students: A cross-sectional study from central India. J Fam Med Prim Care. 2018;7:147–51. 10.4103/jfmpc.jfmpc_189_17. 10.4103/jfmpc.jfmpc_189_17 PMC5958557 29915749

[31] Deb N, Roy P. Internet addiction, depression, anxiety and stress among first year medical students after COVID-19 lockdown: A cross sectional study in West bengal, India. J Fam Med Prim Care. 2022;11:6402–6. 10.4103/jfmpc.jfmpc_809_22. 10.4103/jfmpc.jfmpc_809_22 PMC9810868 36618151

[32] Buckner E. Access to higher education in egypt: examining trends by university sector. Comp Educ Rev. 2013;57:527–52. 10.1086/670665.

[33] Headar MM, Elaref N, Yacout OM. Antecedents and consequences of student satisfaction with e-Learning: the case of private universities in Egypt. J Mark High Educ. 2013;23:226–57. 10.1080/08841241.2013.867919.

[34] Cantini D. We take care of our students: private universities and the politics of care in egypt**. Ethics Soc Welf. 2017;11:261–76. 10.1080/17496535.2017.1300304.

[35] Barsoum G. Quality, pedagogy and governance in private higher education institutions in Egypt. Afr Educ Rev. 2017;14:193–211. 10.1080/18146627.2016.1224558.

[36] Koly KN, Sultana S, Iqbal A, Dunn JA, Ryan G, Chowdhury AB. Prevalence of depression and its correlates among public university students in Bangladesh. J Affect Disord. 2021;282:689–94. 10.1016/j.jad.2020.12.137. 33445093 10.1016/j.jad.2020.12.137

[37] Shafiq S, Nipa SN, Sultana S, Rahman MR-U-, Rahman MM. Exploring the triggering factors for mental stress of university students amid COVID-19 in bangladesh: A perception-based study. Child Youth Serv Rev. 2020;120:105789. 10.1016/j.childyouth.2020.105789. 33518863 10.1016/j.childyouth.2020.105789 PMC7837045

[38] Gupchup GV, Borrego ME, Konduri N. The impact of student life stress on health related quality of life among Doctor of pharmacy students. Coll Stud J. 2004;38:292–302.

[39] Pacheco JPG, Silveira JB, Ferreira RPC, Lo K, Schineider JR, Giacomin HTA, et al. Gender inequality and depression among medical students: A global meta-regression analysis. J Psychiatr Res. 2019;111:36–43. 10.1016/j.jpsychires.2019.01.013. 30665010 10.1016/j.jpsychires.2019.01.013

[40] Gallagher CT, Mehta ANV, Selvan R, Mirza IB, Radia P, Bharadia NS, et al. Perceived stress levels among undergraduate pharmacy students in the UK. Curr Pharm Teach Learn. 2014;6:437–41. 10.1016/j.cptl.2014.02.004.

[41] Elsawy WIH, Sherif AAR, Attia MSED, El-Nimr NA. Depression among medical students in alexandria, Egypt. Afr Health Sci. 2020;20:1416. 10.4314/ahs.v20i3.47. 33402990 10.4314/ahs.v20i3.47 PMC7751550

[42] Stress in health. professions students: myth or reality? A review of the existing literature - PubMed. https://pubmed.ncbi.nlm.nih.gov/16255316/. Accessed 1 Nov 2024. 16255316

[43] Tempski P, Santos IS, Mayer FB, Enns SC, Perotta B, Paro HBMS, et al. Relationship among medical student resilience, educational environment and quality of life. PLoS ONE. 2015;10:e0131535. 10.1371/journal.pone.0131535. 26121357 10.1371/journal.pone.0131535 PMC4486187

[44] Payakachat N, Gubbins PO, Ragland D, Flowers SK, Stowe CD. Factors associated with Health-Related quality of life of student pharmacists. Am J Pharm Educ. 2014;78:7. 10.5688/ajpe7817. 24558275 10.5688/ajpe7817 PMC3930255

